# Texture Analysis of Breast DCE-MRI Based on Intratumoral Subregions for Predicting HER2 2+ Status

**DOI:** 10.3389/fonc.2020.00543

**Published:** 2020-04-21

**Authors:** Hecheng Lu, Jiandong Yin

**Affiliations:** ^1^School of Medicine and Bioinformatics Engineering, Northeastern University, Shenyang, China; ^2^Department of Radiology, ShengJing Hospital of China Medical University, Shenyang, China

**Keywords:** breast cancer, receiver operating characteristic, immunohistochemistry, magnetic resonance imaging, gene expression

## Abstract

**Background:** Breast tumor heterogeneity is related to risk factors that lead to aggressive tumor growth; however, such heterogeneity has not been thoroughly investigated.

**Purpose:** To evaluate the performance of texture features extracted from heterogeneity subregions on subtraction MRI images for identifying human epidermal growth factor receptor 2 (HER2) 2+ status of breast cancers.

**Materials and Methods:** Seventy-six patients with HER2 2+ breast cancer who underwent dynamic contrast-enhanced magnetic resonance imaging were enrolled, including 42 HER2 positive and 34 negative cases confirmed by fluorescence *in situ* hybridization. The lesion area was delineated semi-automatically on the subtraction MRI images at the second, fourth, and sixth phases (P-1, P-2, and P-3). A regionalization method was used to segment the lesion area into three subregions (rapid, medium, and slow) according to peak arrival time of the contrast agent. We extracted 488 texture features from the whole lesion area and three subregions independently. Wrapper, least absolute shrinkage and selection operator (LASSO), and stepwise methods were used to identify the optimal feature subsets. Univariate analysis was performed as well as support vector machine (SVM) with a leave-one-out-based cross-validation method. The area under the receiver operating characteristic curve (AUC) was calculated to evaluate the performance of the classifiers.

**Results:** In univariate analysis, the variance from medium subregion at P-2 was the best-performing feature for distinguishing HER2 2+ status (AUC = 0.836); for the whole lesion region, the variance at P-2 achieved the best performance (AUC = 0.798). There was no significant difference between the two methods (*P* = 0.271). In the machine learning with SVM, the best performance (AUC = 0.929) was achieved with LASSO from rapid subregion at P-2; for the whole region, the highest AUC value was 0.847 obtained at P-2 with LASSO. The difference was significant between the two methods (*P* = 0.021).

**Conclusion:** The texture analysis of heterogeneity subregions based on intratumoral regionalization method showed potential value for recognizing HER2 2+ status in breast cancer.

## Introduction

Breast cancer is the most common malignant tumor in women worldwide. Molecular subtypes of breast cancer, which are indicators of disease-free and overall survival, can be used to guide targeted therapy (1, 2). A classification system based on tumor genotype categorizes breast tumors into four molecular subtypes: luminal A, luminal B, human epidermal growth factor receptor 2 (HER2)-overexpressing, and basal-like (3–6). HER2 is a cell-surface receptor that is present in normal mammary gland cells and controls growth, division, and repair of breast cells (7, 8). HER2-positive tumors account for 20–25% of human breast tumors (9). HER2-overexpressing cancers are characterized by rapid growth and division of tumor cells, promoting cell proliferation and angiogenesis (10, 11). HER2-positive breast cancers are associated with a worse survival, a poorer prognosis, and a higher risk of recurrence than HER2-negative cases; however, they are more sensitive to neoadjuvant trastuzumab-based therapy (12–14). Thus, it is critical to identify the HER2 status of breast cancer to select the appropriate treatment and evaluate the response to therapy. HER2 status is generally detected using streptavidin-peroxidase staining for immunohistochemistry (IHC) analysis. IHC staining for HER2 is scored from 0 to 3+: scores of 0 and 1+ are considered HER2-negative, and a score of 3+ is considered HER2-positive. Cases of HER2 2+ need to be analyzed by fluorescence *in situ* hybridization (FISH) to confirm the expression status (15–17). However, the determination of HER2 2+ status by FISH is expensive, time consuming, and requires specialized equipment and technical skills. Therefore, there is an urgent demand for the development of a sensitive, quick, easy-to-use, and cost-effective alternative method to identify HER2 2+ status.

Due to tumor growth, heterogeneity is caused by the new immature, tortuous and hyper-permeable capillaries from the existing blood vessels, and found in many breast carcinomas (18–21). Dynamic contrast-enhanced magnetic resonance imaging (DCE-MRI) is currently considered the most sensitive imaging modality for assessing microvessel distribution and blood perfusion in breast cancer (22, 23). Studies have used the heterogeneity of breast DCE-MRI images within the whole tumor to build prediction models of tumor subtypes (24, 25). A recent study investigated the association between Oncotype DX RS and DCE-MRI texture features (26). A related study by Eric et al. analyzed the DCE-MRI kinetic characteristics by quantifying the percent volume of the tumor, which is associated with HER2 status (27).

These studies based on DCE-MRI data provide useful information by quantifying heterogeneity in the entire tumor. However, analysis of intratumoral regions may provide valuable clues that could be missed in the analysis of whole tumors (28, 29). Previous studies analyzing the texture features from intratumoral regions on DCE-MRI mainly focused on predicting the pathological response of breast cancer to neoadjuvant chemotherapy (30–32). Few studies have used the texture features from tumor subregions for predicting the molecular subtypes of breast cancer (24). To the best of our knowledge, there are no studies investigating the association between texture features extracted using the intratumoral regionalization method and HER2 2+ status of breast cancers.

## Materials and Methods

### Patient Cohort

The study was approved by the Ethics Committee of Shengjing Hospital of China Medical University (NO.2019PS175K). All images were retrospectively selected after removing all patient information; therefore, the requirement for informed consent was waived. The study enrolled 465 patients with pathologically confirmed breast cancer who underwent DCE-MRI between November 2017 and August 2018. Patients were excluded if the following conditions were met: (1) cases with HER2 scores of 0, 1+, and 3+ verified by IHC (*n* = 278); (2) cases with HER2 2+ not tested by FISH (*n* = 64); and (3) cases treated with chemotherapy or radiation therapy before MRI examination (*n* = 47). Finally, 76 patients with HER2 2+ status verified by FISH were selected for subsequent analysis.

### MR Image Acquisition

All DCE-MRI examinations were performed with a GE 3.0T MRI scanner (Signa HDxt, GE Healthcare, USA), and each patient was scanned in the prone position using a dedicated eight-channel double-breast coil. The orientation of slice images was transverse. For each MRI scan, a pre-contrast series of VIBRANT-VX sequence T1-weighted 3D images (mask images) was initially acquired. Eight post-contrast scans were performed after intravenous injection of the contrast agent (0.5 mmol/mL, Gadodiamide, Omniscan, GE Healthcare, USA; Magnevist, Bayer-Schering Pharmaceuticals) at 4 mL/s (0.15 mmol/kg body weight) and an equal volume of saline flush at the same injection speed. The DCE-MRI scanning parameters were as follows: repetition time = 7.42 ms, echo time = 4.25 ms, flip angle = 15, slice thickness = 2.20 mm, spacing between slices = 2.20 mm, inversion time = 20 ms, image matrix = 1024 × 1024, temporal acquisition = 80 s, and slice number = 78.

### Research Framework

**Figure 1:**
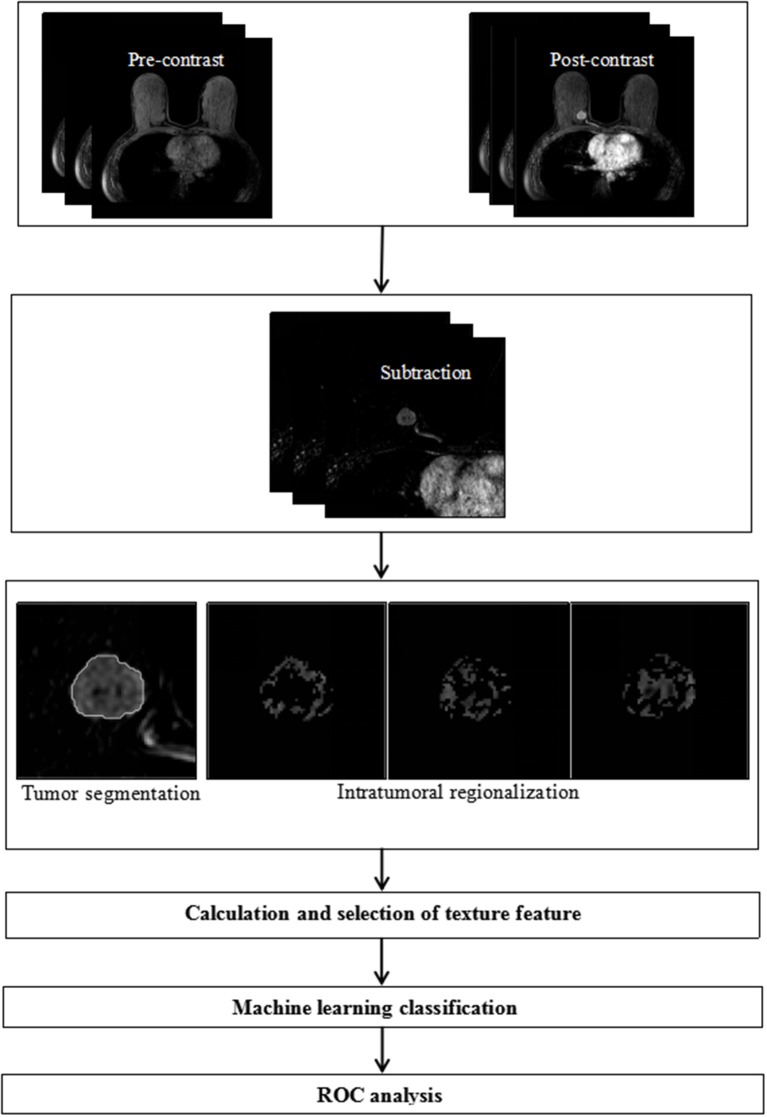
Flowchart of the proposed method for discriminating HER2 2+ status.

This study was performed in several stages as follows: first, the lesion area of each case was extracted semi-automatically based on the subtraction DCE-MRI images. Second, each lesion was divided into three subregions by an intratumoral partitioning method according to the time to peak (TTP) of the kinetic curve. Third, texture features were extracted from both lesion areas and subregions separately. Fourth, three feature selection methods were used to generate optimal feature subsets. Fifth, a logistic regression model was applied to classify tumors with different HER2 2+ status. Finally, receiver operating characteristic (ROC) analysis was performed to evaluate the performance of the model. The processing of tumor segmentation, intratumoral regionalization, feature extraction and selection was performed using the Matlab programming platform (version R2018a, Mathworks, Natick, MA). Figure 1 shows the flowchart of the classification system.

### Tumor Segmentation

For each patient, eight subtraction volume images were obtained by subtracting pre-contrast images from post-contrast images. All slice images (8 volume × 78 slice images/per volume = 624 slice images) were read by two experienced radiologists, and the slice image with optimal representation of the largest tumor area was selected with an evaluation consensus between the two radiologists for subsequent lesion segmentation, as well as those from the same slice at the other seven phases. Because of the exploratory nature of the study, lesion segmentation was only performed on slices extracted at the second, fourth, and sixth phases (termed P-1, P-2, and P-3, respectively). Arbitrarily shaped regions of interest containing the lesion area were independently drawn by two radiologists with more than 10 years of experience in reading breast MRI images who was blinded to the pathological diagnosis. As reported in previous studies, the lesion boundary was automatically delineated by a spatial fuzzy C-means algorithm and was then refined using morphological processing (33–35). The results of semi-automatic segmentation were examined and corrected by another radiologist with 12 years of experience in interpreting breast MRI.

### Intratumoral Regionalization

To survey intratumoral heterogeneity, a previously reported method was used to divide the lesion area into three subregions according to the variation of pixel intensity at different imaging phases (24). Briefly, the method used was as follows:

First, the relative enhancement was calculated by comparing post-contrast images with pre-contrast images on a pixel-by-pixel basis using the following equation.

$$\begin{array}{l} E\left(x,y,t\right)=\frac{SI\left(x,y,t\right)-SI\left(x,y,t\right)}{SI\left(x,y,t_{0}\right)}\times 100\%,t=\left\{1,2,3\ldots 8\right\} \end{array}$$

where *E*(*x, y, t*) represents the percentage of enhancement, *SI*(*x, y, t*) represents the intensity of pixels (*x, y*) measured at time *t*, and *t*_0_ is the precontrast time instant. The kinetic curve, *E*(*x, y, t*), was defined for describing the change of relative enhancement over time (36). TTP, the time at which peak enhancement was achieved, was computed from the kinetic curve as follows.

$$\begin{array}{l} TTP\left(x,y\right)=\arg\max\underset{t}{E}\left(x,y,t\right) \end{array}$$

We then partitioned the pixels within the lesion area based on their TTP values. More specifically, the lesion pixels at the first four, fifth or sixth, and seventh or eighth phases for reaching peak enhancement values were grouped into rapid, medium, and slow subregions, respectively. Therefore, the whole lesion was partitioned into three subregions representing various extensions of TTP values.

### Feature Extraction and Selection

Texture features were extracted from the lesion area and subregions to determine the heterogeneity of breast tumors. A total of 488 texture features were measured, including histogram-based, gray-level co-occurrence matrix (GLCM)-based, gray-level run-length matrix (GRLM)-based, and discrete wavelet transform (DWT)-based features, as shown in Table 1.

**Table 1:** Features measured with different texture analysis methods.

**Methods**	**Texture features**	**Number**
Histogram	Mean, variance, skewness, kurtosis	4
GLCM^a^	Autocorrelation (ACOR), contrast (CON), correlation (COR), cluster prominence(CP), cluster shade (CS), dissimilarity (DIS), energy (ENE), entropy (ENT), homogeneity (HOM), maximum probability (MP), sum of squares (SOS), sum average (SA), sum variance (SV), sum entropy (SE), difference variance (DV), difference entropy (DE), information measure of correlation (IMC), inverse difference normalized (IDN), inverse difference moment normalized (IDMN)	380
GRLM^b^	Run-length non-uniformity (RLN), gray level non-uniformity (GLN), long run emphasis (LRE), short run emphasis (SRE), fraction of image in runs (FIR), low gray-level run emphasis (LGRE), high gray-level run emphasis (HGRE), short run low gray-level emphasis (SRLGE), short run high gray-level emphasis (SRHGE), long run low gray-level emphasis (LRGE), long run high gray-level emphasis(LRGE)	44
DWT^c^	Harr parameters	20
	Deubechies2 parameters	20
	Symlet4 parameters	20
Total		488

*GLCM, gray level co-occurrence matrix; GRLM, gray level run-length matrix; DWT, discrete wavelet transformation*.

a *GLCM parameters were calculated for four distances (1, 2, 3, and 4 pixels) and four angles (0, 45, 90, and 135°). (d, 0), (0, d), (d, d), (–d, –d) represent 0, 45, 90, and 135°, respectively, where d is the distance. For example, CON (0, 1) represents the contrast feature calculated for a distance of 1 and a direction of 90°*.

b *GELM parameters were calculated for four angles (0, 45, 90, and 135°)*.

c *DWT parameters were calculated for four layers and three directions (horizontal, vertical, diagonal) to produce low and high frequency components. For example, Haar HD_2 represents the diagonal high frequency component of the second layer using the Haar wavelet*.

Feature filter is a crucial step for building robust learning models by removing most irrelevant and redundant features from the entire feature set. In particular, the feature selection is even more important in the analysis of high-dimensional datasets with the number of features largely exceeding the number of observations (37). In this study, the feature selection procedure was as follows:

1) Features with small variance values (i.e., a variance value < 0.01) were removed to eliminate meaningless features (38, 39).
2) Features with high similarity (i.e., a Pearson correlation coefficient > 0.95 with other features) were removed to reduce colinearity features (40).

### Statistical Analysis

Differences in categorical variables were assessed using the *x*^2^ test or Fisher's exact test when the expected value in any cell of the table was < 5. A univariate logistic regression classifier was used to assess the performance of individual features for differentiating HER2 2+ status. A prevalent supervised learning model, support vector machine (SVM), was applied to classify expression status of HER2 2+. To avoid overfitting of classifiers, a leave-one-out cross-validation (LOOCV) method was used (41). In each loop of the LOOCV, one sample was retained as the test case, and the other samples were used as the training set. At each LOOCV loop, three feature selection methods, wrapper, least absolute shrinkage and selection operator (LASSO), and stepwise, were, respectively, applied on the training set. The procedure was repeated for all LOOCV folds, and a classification score was generated for each test case. The importance of image features was evaluated by counting the number of times they were selected over all of the LOOCV loops.

To assess the performance of classifiers in distinguishing HER2 2+ status, a ROC curve was drawn using the professional statistics software MedCalc (version 14.10.20, http://www.medcalc.org/), and the area under the ROC curve (AUC) was calculated as an indicator of diagnostic performance. The sensitivity, specificity and accuracy were also determined. The *z*-test was used to evaluate the statistical significance of differences between AUCs (42).

The intraobserver variability of features extracted by two radiologists was evaluated using intraclass correlation coefficients (ICCs, 0–0.4, poor agreement; 0.41–0.6, moderate agreement; 0.61–0.8, good agreement; 0.81–1, excellent agreement) (43, 44).

A *P*-value < 0.05 was considered statistically significant, and statistical analyses were performed using SPSS software (version 19.0, Chicago, IL, USA).

## Results

### Subjects

**Figure 2:**
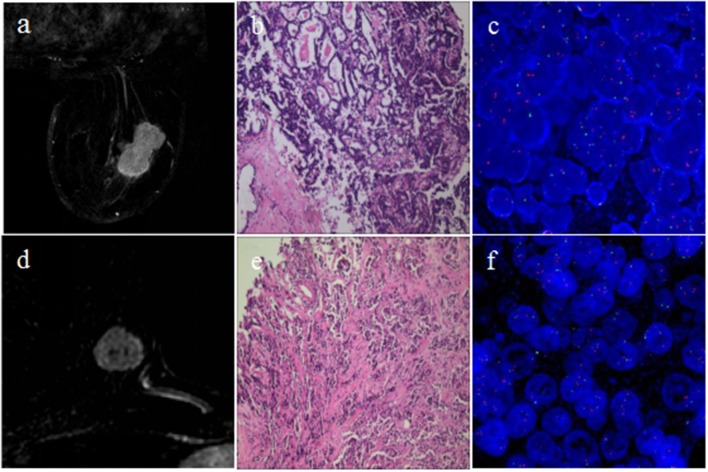
Cases of breast cancers with different HER2 2+ status. The first row shows one case with positive HER2 2+ status; **(a)** subtraction MRI image at P-2; **(b)** pathology result showing invasive carcinoma of no special type; **(c)** FISH result showing positive HER2 status. The second row shows the other case with negative HER2 2+ status; **(d)** subtraction MRI image at P-2; **(e)** pathology result showing invasive carcinoma of no special type; **(f)** FISH result showing negative HER2 status.

**Figure 3:**
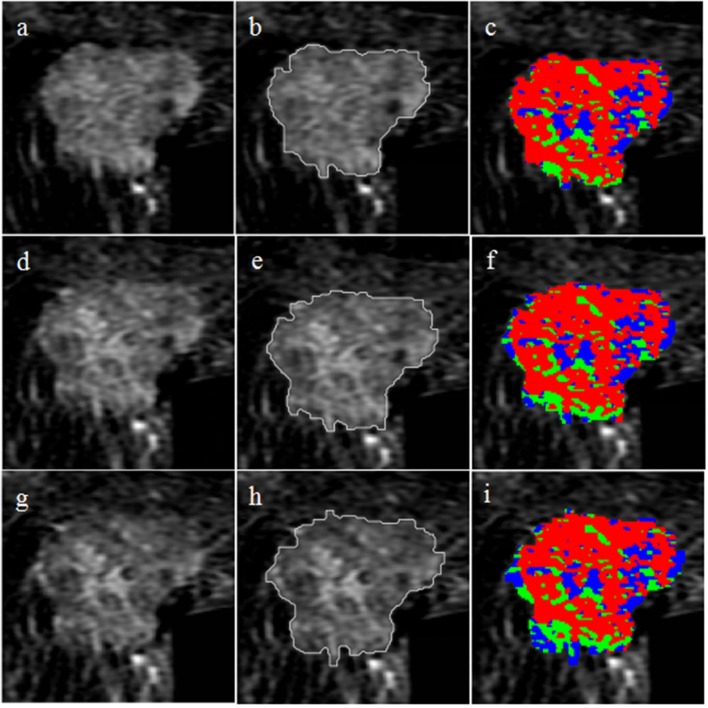
Tumor segmentation and intratumoral regionalization results for a randomly selected case. The first row shows the results at P-1; **(a)** axial T1-weighted fat-saturated subtraction MRI image; **(b)** tumor segmentation result obtained with the semi-automatic method; **(c)** intratumoral regionalization result where the red, green, and blue colors represent rapid, medium, and slow subregions, respectively. The second row shows the results at P-2; **(d)** subtraction MRI image; **(e)** tumor segmentation result; **(f)** intratumoral regionalization result. The third row shows the results at P-3; **(g)** subtraction MRI image; **(h)** tumor segmentation result; **(i)** intratumoral regionalization result.

The selected 76 cases included 42 (55.3%) HER2 2+ positive and 34 (44.7%) negative patients. Figure 2 shows two representative cases, one with positive HER2 status and one with negative HER2 status. The detailed characteristics of the 76 cases are presented in Table 2. There was no significant association between HER2 2+ status and patient characteristics. A randomly selected case (HER2 status, positive) is shown in Figure 3 to illustrate the results of tumor segmentation and intratumoral regionalizaton based on the method proposed above.

**Table 2:** Characteristics of 76 patients with HER2 2+ breast cancer.

**Characteristics**	**FISH Results**		***P*-Value^a^**
	**Positive (*n* = 42) (%)**	**Negative (*n* = 34) (%)**	
**Age**^b^			0.846
≥40 years at diagnosis	34 (56.7)	26 (43.3)	
<40 years at diagnosis	8 (50.0)	8 (50.0)	
**Progesterone receptor**^b^			0.531
Positive	34 (53.1)	30 (46.9)	
Negative	8 (66.7)	4 (33.3)	
**Ki-67**^**2**^			0.594
≥14%	33 (57.9)	24 (42.1)	
<14%	9 (55.3)	10 (44.7)	
**Pathology**^c^			0.658
Ductal carcinoma *in situ*	1 (50.0)	1 (50.0)	
invasive carcinoma of no special type	40 (54.8)	33 (45.2)	
Invasive microcapillary carcinoma	1 (100)	0 (0)	

a *P-value is for positive-HER2 vs. negative-HER2 comparison*.

b *Variables are tested using the x^2^ test*.

c *Variables are tested using Fisher's exact test*.

*Numbers in parentheses represent percentages*.

### Univariate Analysis

**Figure 4:**
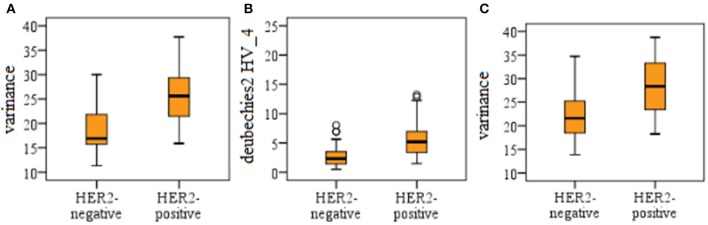
Boxplot graphs for the three individual features with the best performance. The three features were variance from the medium region at P-2 **(A)**, deubechies2 HV_4 from rapid region at P-1 **(B)**, and variance from the slow region at P-3 **(C)**, respectively.

For univariate regression analysis, the top eight individual features with the best performance according to AUC values are listed in Table 3 for the whole lesion region and three subregions. The results demonstrated that the ability of a single feature from the whole lesion area was relatively lower than that from subregions for distinguishing HER2 2+ status. The rapid regions showed the best performance, and the best eight individual features had AUC values ranging from 0.773 to 0.810. The best single feature was variance from the medium subregion at P-2, which achieved an AUC value of 0.836. There was no significant difference comparing with that feature from the whole lesion in the diagnostic performance (*P* = 0.271). The best individual features from rapid and slow subregions were deubechies2 HV_4 at P-1 (AUC = 0.810) and variance at P-3 (AUC = 0.807), respectively. For the best three individual features, the differences between positive and negative HER2 status is shown in Figure 4 using boxplot graphs.

**Table 3:** Univariate analysis for predicting HER2 2+ status.

**Method**	**Subregions**	**Features**	**AUC**	**Interval**	***P*-value^a^**
Intratumoral Regionalization	Rapid	deubechies2HV_4(P-1)	0.810	0.722-0.912	0.0001
		deubechies2HV_4(P-2)	0.787	0.686-0.888	0.0002
		mean(P-1)	0.785	0.681-0.889	0.112
		mean(P-2)	0.784	0.683-0.884	0.438
		0°LRE(P-2)	0.777	0.672-0.881	0.004
		deubechies2HV_4(P-3)	0.775	0.672-0.879	0.012
		0°LRE(P-3)	0.774	0.667-0.881	0.008
		variance(P-2)	0.773	0.666-0.881	0.531
	Medium	variance(P-2)	0.836	0.748-0.932	0.271
		variance(P-3)	0.796	0.695-0.896	0.191
		variance(P-1)	0.769	0.661-0.878	0.542
		mean(P-2)	0.764	0.958-0.872	0.786
		mean(P-1)	0.748	0.636-0.861	0.681
		mean(P-3)	0.723	0.607-0.841	0.846
		45°RLN(P-1)	0.676	0.553-0.799	0.024
		45°RLN(P-3)	0.673	0.551-0.796	0.013
	Slow	variance(P-3)	0.807	0.708-0.901	0.112
		variance(P-2)	0.802	0.697-0.903	0.973
		variance(P-1)	0.782	0.678-0.885	0.823
		mean(P-2)	0.747	0.638-0.856	0.289
		mean(P-3)	0.731	0.615-0.844	0.893
		harr HD_3(P-1)	0.711	0.596-0.826	0.143
		mean(P-1)	0.703	0.583-0.824	0.071
		symlet4 HH_3(P-1)	0.672	0.546-0.795	0.715
Whole lesion	/	variance(P-2)	0.798	0.699-0.898	/
		variance(P-1)	0.789	0.688-0.895	/
		mean(P-2)	0.771	0.666-0.873	/
		variance(P-3)	0.762	0.654-0.872	/
		mean(P-1)	0.758	0.648-0.869	/
		mean(P-3)	0.727	0.614-0.844	/
		45°GLN(P-1)	0.713	0.597-0.829	/
		deubechies2 L_2(P-3)	0.712	0.598-0.827	/

a *P-values indicate performance comparisons between features from intratumoral subregions and the same types of features from the whole lesion area at the same phase*.

### SVM Analysis

**Figure 5:**
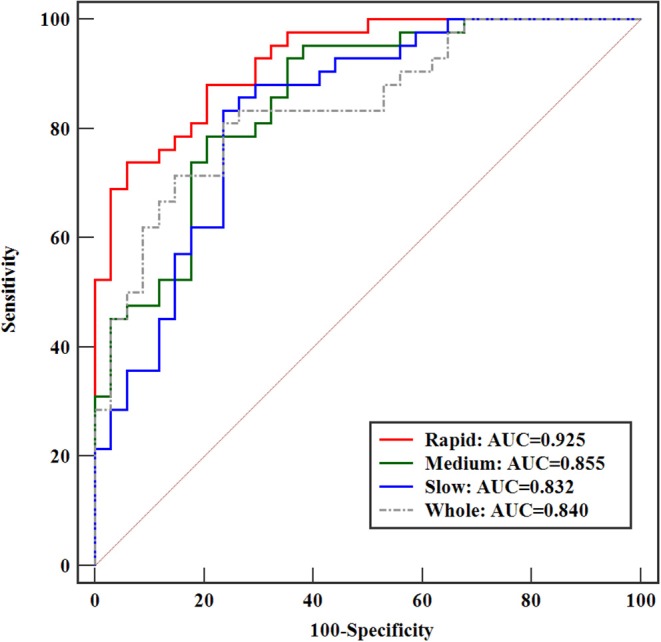
Comparison of ROCs among the best-performance classification models for the whole lesion and three subregions.

Further classification assessment was performed for identifying HER2 2+ status using the SVM model. The final results are shown in Table 4. In the rapid subregions, the classifier produced an AUC value of 0.929, which was the best performance (88.16% accuracy, 88.10% sensitivity, and 88.24% specificity) among the three subregions. The performance was significantly higher (*P* = 0.026) than that of the whole lesion area (AUC = 0.847). The best performance was achieved at P-2, and 13 texture features were selected by the LASSO method, as shown in Table 5. The prediction models in medium and slow subregions reached AUCs of 0.855 and 0.834, respectively, which was higher than the values obtained with the model based on the whole tumor. The comparisons of the ROCs among the best classification models for whole lesion and three subregions are shown in Figure 5. The comparisons among the three feature selection methods achieving the best classification performances are outlined in Table 6. Taken together, the results indicate that the rapid subregions achieved a higher performance for HER2 2+ classification than other subregions.

**Table 4:** Multivariate analysis for predicting HER2 2+ status.

**Methods**			**Subregions**			**Whole lesion**
			**Rapid**	**Medium**	**Slow**	
P-1	PCA	AUC	0.847	0.793	0.721	0.697
		*P*-value^a^	0.008	0.034	0.686	/
	LASSO	AUC	0.905	0.854	0.832	0.835
		*P*-value	0.062	0.613	0.906	/
	stepwise	AUC	0.901	0.543	0.751	0.797
		*P*-value	0.053	0.001	0.426	/
P-2	PCA	AUC	0.869	0.803	0.751	0.821
		*P*-value	0.371	0.761	0.213	/
	LASSO	AUC	0.925	0.855	0.825	0.840
		*P*-value	0.026	0.701	0.727	/
	stepwise	AUC	0.917	0.665	0.746	0.806
		*P*-value	0.012	0.021	0.281	/
P-3	PCA	AUC	0.876	0.736	0.758	0.714
		*P*-value	0.005	0.723	0.397	/
	LASSO	AUC	0.861	0.791	0.826	0.739
		*P*-value	0.024	0.221	0.113	/
	stepwise	AUC	0.842	0.543	0.809	0.723
		*P*-value	0.047	0.011	0.143	/

a *P-values indicate performance comparisons between features from subregions and the whole lesion area*.

**Table 5:** Thirteen texture features selected by the LASSO method from rapid subregions at P-2.

**Features**	**FISH**	
	**Positive**	**negative**
mean	100.668 ± 27.003	70.911 ± 26.085
variance	23.910 ± 7.587	17.923 ± 6.298
ACOR (0,1)	3.201 ± 1.663	2.025 ± 0.918
IMC (-1,-1)	−0.482 ± 0.096	−0.393 ± 0.148
haar HH_2	6.604 ± 2.522	7.908 ± 3.586
symlet4 HD_2	3.302 ± 1.753	4.691 ± 2.527
haar L_4	35.696 ± 16.721	23.261 ± 16.893
haar HH_3	4.933 ± 2.053	5.187 ± 2.799
haar HD_4	2.633 ± 1.638	2.504 ± 1.755
deubechies2 HH_4	4.126 ± 1.679	4.109 ± 3.113
deubechies2 HD_3	5.469 ± 2.369	6.093 ± 4.011
symlet4 HH_3	4.712 ± 2.355	5.144 ± 2.534
symlet4 L_4	5.875 ± 5.280	4.696 ± 3.475

*Values represent the mean±standard deviation*.

**Table 6:** Comparison among the three feature selection methods achieving the best classification performances.

**Method**	**AUC**	**Sensitivity (%)**	**Specificity (%)**	**Accuracy (%)**
PCA	0.876 (rapid region at P-3)	80.95	82.35	81.58
LASSO	0.925 (rapid region at P-2)	73.81	94.12	82.92
Stepwise	0.917 (rapid region at P-2)	76.19	93.26	84.21

### Interobserver Variability Evaluation

The texture features derived from the two sets of ROIs delineated independently by two radiologists showed excellent agreement (ICCs, 0.867–0.932).

### Discussion

In this work, an intratumoral partitioning method was used to divide the lesion area into different subregions in which texture features reflecting the tumor heterogeneity were computed for predicting HER2 2+ status of breast cancers. A significant correlation between texture features and HER2 2+ status was found, and the features extracted from the rapid subregion achieved the best performance. The present findings indicate that the texture features extracted from intratumoral subregions have potential value for distinguishing the HER2 2+ status of breast cancer.

Intratumoral heterogeneity is an indicator of differences in gene expression, angiogenesis, metabolism, and other biological characteristics (45–47). Previous studies explored heterogeneity based on DCE-MRI texture features from the whole lesion area (25, 48). However, intratumoral heterogeneity in subregions has not been investigated thoroughly in breast cancer. In this study, an effective intratumoral partitioning method was applied to divide the lesion area into three subregions for predicting HER2 2+ status. The prediction model with the highest performance was established on the rapid subregions. One possible reason for this result is that the rapid subregions may be more closely related to angiogenesis, which was considered as the consequence of HER2 2+ amplification.

In recent years, most studies used GLCM texture features for predicting the molecular subtypes of breast tumors or the pathological response to neoadjuvant chemotherapy (49, 50). In this study, additional features were explored, such as histogram features, GRLM features, and DWT features, and the experimental results of univariate analysis demonstrated that those features were highly related to HER2 2+ expression status. The individual texture feature, namely, variance from medium subregions at P-2, was the best-performing individual feature for differentiating HER2 2+ status. The variance feature is an indicator for measuring the degree of dispersion. If the variance value is high, the signal intensity of the image is dispersive (51). Therefore, the variance feature could be considered as a marker reflecting the abnormal architecture of a heterogeneous tumor. Additionally, the feature deubechies2 HV_4 from rapid subregions at P-1 showed significantly better (*P* = 0.0001) performance in terms of the AUC value than that extracted from the whole tumor. DWT parameters representing the image signal in different frequencies and directions showed a relatively high performance, which was not reported in previous relevant studies.

In multivariate analysis, three feature-dimension reduction methods (PCA, LASSO, and stepwise) were used to select the most useful features from the whole feature set. In the PCA, the best performance was achieved on the rapid region at P-3, and eight principal components with a total contribution score > 95% were derived. The optimal performance derived from the PCA method was statistically significantly different from that of the whole lesion (*P* = 0.005). Compared with PCA, the LASSO method with an interpretable model showed the best performance among all methods tested in this study, and 13 features were finally selected from the rapid region at P-2. In the stepwise method, the maximum *P*-value derived from the *F*-statistic test for adding a feature was set to 0.05, and the minimum *P*-value for removing a feature was set to 0.1 (52). The stepwise method applied to rapid and slow regions showed a better performance (AUC values 0.746–0.917) than that applied to medium regions (AUC values 0.543–0.665).

We evaluated the intraobserver variability for texture features extracted from the whole lesion region and three different intratumoral sub-regions at the three imaging time points. The results showed high consistency between two radiologists regarding the calculation of texture features based on the manual method, with ICCs ranging from 0.867 to 0.932. Intraobserver variability was mostly associated with slice selection and delineation of the ROI, because the next step was calculating texture features within the ROI using in-house software with MATLAB 2018a. This means that the methods used for ROI definition are important.

This work performed a preliminary analysis of intratumoral features for identifying the HER2 2+ status of breast cancer. However, the study had several limitations. First, the relatively small sample size limited the statistical power of the study. Therefore, additional samples must be collected to confirm and refine the present results in future work. Second, only a single representative slice was applied to extract the tumor features in this study. Texture analysis based on 3D volume images might be one of the strategies to improve the classification performance in identifying gene expression status of HER2 2+ (53). Relatively, analysis based on one single slice would inevitably lose a lot of important information because of heterogeneity in tumor volume. Hence, some previous studies conducted the 3D segmentation for radiomic analysis (11, 54–56). It must be pointed out that in order to achieve 3D segmentation, the image data should be acquired using the isotropic acquisition when the cases were initially scanned. Actually, that is not easy to achieve with complete breast coverage without significantly degrading the temporal resolution. For anisotropic data, a more true representation of the lesion can be obtained with multi-slice 2D segmentation than obtained with a single slice analysis (57). Third, we only analyzed the conventional methods for feature calculation. Some more advanced features, which many ongoing studies have focused, were not investigated in discriminating HER2 2+ status, such as Scale Invariant Feature Transform (SIFT), Speeded-Up Robust Features (SURF), Histogram of Oriented Gradient (HOG), Local Binary Pattern (LBP), Local Self Similarity (LSS) (58–60). Meanwhile, because each kind of feature has its own limitations, a consensus was reached that no single feature has a perfect performance in radiomic analysis. Considerable attention has been received from researchers on how to fuse those advanced features for improving the classification performance (61). In addition, due to the variety of features, feature selection methods for establishing predictive model has received more and more attention in machine learning selection. Therefore, in order to further improve the diagnosis efficiency and robustness in the determination of HER2 2+ status, it is necessary to validate more advanced approaches in feature calculation, selection and fusion. Finally, only texture features were investigated in DCE-MRI images, whereas other types of quantitative parameters from different imaging modalities, such as intra-voxel incoherent motion diffusion weighted imaging (IVIMDWI), diffusion weighted imaging (DWI) and diffusion tensor imaging (DTI), were not explored, which might be useful for HER2 2+ status discrimination.

In conclusion, texture analysis based on an intratumoral regionalization method for breast DCE-MRI could be used to predict HER2 2+ status. Further studies with a larger sample size and more quantitative features should be performed to improve the accuracy of the results.

## Data Availability Statement

The datasets analyzed in this article are not publicly available as the data contain potentially identifying or sensitive patient information. Requests to access the datasets should be directed to Jiandong Yin, jiandongyin@sina.com.

## Ethics Statement

The studies involving human participants were reviewed and approved by Shengjing Hospital of China Medical University. The ethics committee waived the requirement of written informed consent for participation. Written informed consent was not obtained from the individual(s) for the publication of any potentially identifiable images or data included in this article.

## Author Contributions

HL did the analysis. JY conducted manuscript writing. All authors critically reviewed and revised the manuscript.

## Conflict of Interest

The authors declare that the research was conducted in the absence of any commercial or financial relationships that could be construed as a potential conflict of interest.
